# P-2178. The Impact of the Respiratory Syncytial Virus (RSV) Vaccination Program: Uptake and Hospitalization Trends in Older Adults in England

**DOI:** 10.1093/ofid/ofaf695.2341

**Published:** 2026-01-11

**Authors:** William Bentley, Madeleine L Smith, Femke M Ahlers, Tristan Curteis

**Affiliations:** Costello Medical, Bristol, England, United Kingdom; Costello Medical, Bristol, England, United Kingdom; Costello Medical, Bristol, England, United Kingdom; Costello Medical, Bristol, England, United Kingdom

## Abstract

**Background:**

Respiratory syncytial virus (RSV) is a common infectious disease which generally presents with mild symptoms; however, severe infections in children, older adults and immunocompromised people cause a substantial number of hospitalizations in England every year. The recent approval and rollout of RSV vaccines for older adults aimed to reduce this disease burden. Here, we examine the rollout of the RSV vaccine among older adults in England from September 2024, investigating vaccine uptake and its impact on hospitalization rates.

**Figure:**
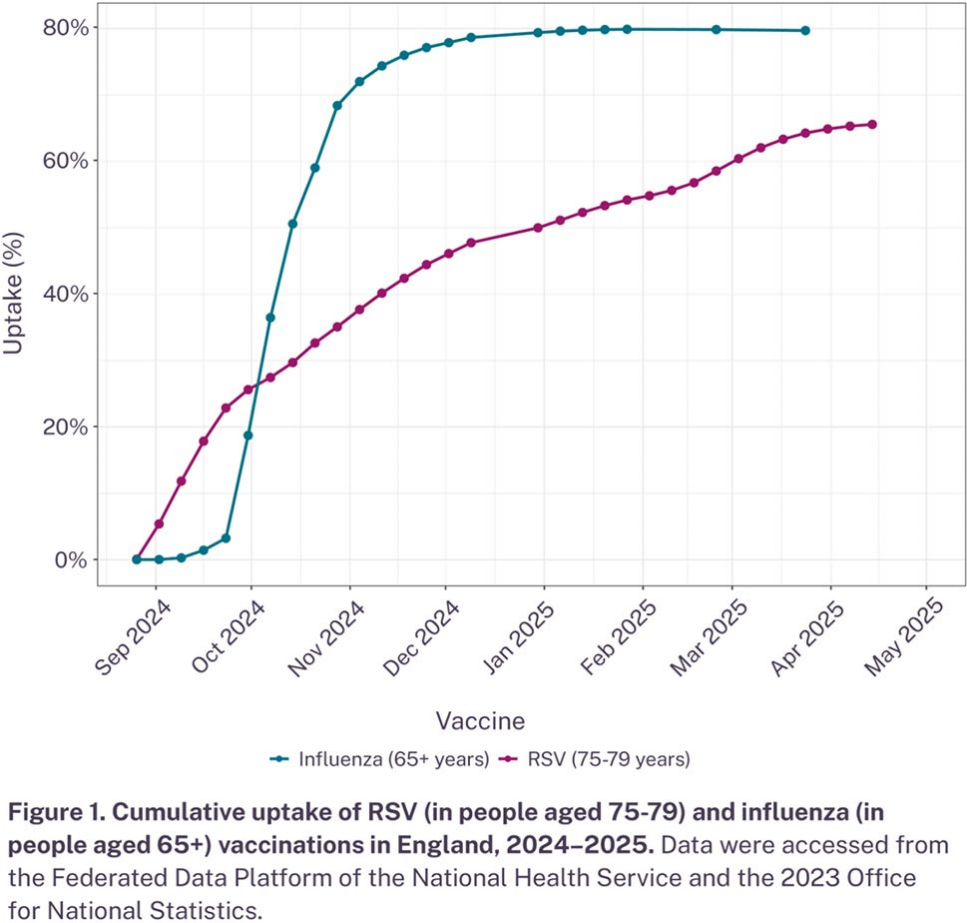

**Figure:**
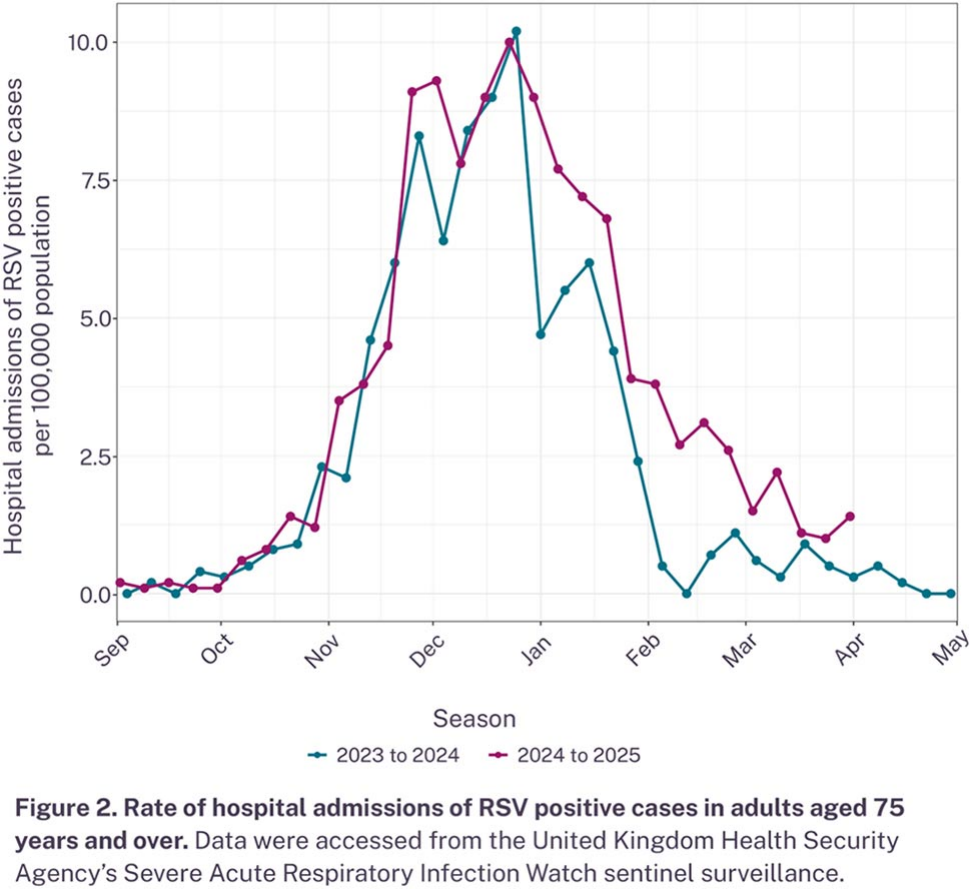

**Methods:**

RSV vaccine uptake for people in England aged 75-79 was calculated for the first 34 weeks of the vaccine rollout period (1^st^ September 2024 to 20^th^ April 2025) using data from the Federated Data Platform of the National Health Service and 2023 Office for National Statistics population data. Hospital admission rates for RSV positive cases for people aged 75 and above were accessed through the United Kingdom Health Security Agency’s Severe Acute Respiratory Infection Watch sentinel surveillance, data from this source was compared for the 2023–2024 to the 2024–2025 season.

**Results:**

Cumulative RSV vaccine uptake in adults aged 75-79 reached 65.6% by April 2025, which was lower than influenza vaccine uptake levels in people aged 65 or above in England (79.7% by March 2025; Figure 1). Hospital admission rates for RSV positive cases for people aged 75 or above in the 2024–2025 season were comparable to the previous year, when an RSV vaccine was not available for the older adult population (Figure 2).

**Conclusion:**

While RSV vaccine uptake this season was below that of the influenza vaccine, uptake would be expected to increase in future seasons with greater awareness of the immunization program. Although an immediate reduction in RSV hospitalizations was not observed in 2024–2025 compared to 2023–2024, continued efforts to increase vaccine uptake rates through improved program visibility and integration with other vaccination programs could maximize benefits. Ongoing surveillance of vaccine uptake and epidemiological monitoring of the disease is essential to understand the long-term impact of the recently rolled out RSV vaccines in England and to improve future vaccination strategies.

**Disclosures:**

William Bentley, MBiochem, Costello Medical: Employee Madeleine L. Smith, PhD, Costello Medical: Employee Femke M. Ahlers, DPhil, Costello Medical: Employee

